# Titanium Oxyfluoride as a Material for Negative Electrodes of Lithium-Ion Batteries

**DOI:** 10.3390/ijms24054968

**Published:** 2023-03-04

**Authors:** Ekaterina V. Astrova, Vladimir P. Ulin, Alesya V. Parfeneva, Galina V. Li, Maria A. Yagovkina, Darina A. Lozhkina, Andrei A. Krasilin, Maria V. Tomkovich, Aleksander M. Rumyantsev

**Affiliations:** Ioffe Institute, Russian Academy of Sciences, Politekhnicheskaya st. 26, 194021 Saint Petersburg, Russia

**Keywords:** Li-ion batteries, negative electrodes, titanium oxyfluoride, cycling stability, CVA measurements, diffusion coefficient, Coulomb efficiency over 100%

## Abstract

A study of the electrochemical characteristics of titanium oxyfluoride obtained with the direct interaction of titanium with hydrofluoric acid is reported. Two materials T1 and T2 synthesized under different conditions in which some TiF_3_ is formed in T1 are compared. Both materials exhibit conversion-type anode properties. Based on the analysis of the charge–discharge curves of the half-cell, a model is proposed according to which the first electrochemical introduction of lithium occurs in two stages: the first stage is the irreversible reaction resulting in a reduction in Ti^4+/3+^, and the second stage is the reversible reaction with a change in the charge state Ti^3+/1.5+^. The difference in material behavior is quantitative: T1 has a higher reversible capacity but lower cycling stability and a slightly higher operating voltage. The Li diffusion coefficient determined from the CVA data for both materials averages 1.2–3.0 × 10^−14^ cm^2^/s. A distinctive feature of titanium oxyfluoride anodes is the asymmetry in kinetic characteristics that revealed themselves during lithium embedding and extraction. In the long cycling regime, the excess of Coulomb efficiency over 100% was found in the present study.

## 1. Introduction

Transition metal oxides and fluorides have the ability to reversibly absorb lithium during lithium-ion battery operation [1]. A characteristic feature of most of these materials is an irreversible conversion reaction under the first lithiation that results in a partial metal reduction with the release of amorphous Li_2_O and/or LiF. Nanoscale precipitates of intermediate compounds containing partially reduced transition metal atoms are formed. Materials undergoing chemical conversion during lithiation/delithiation typically exhibit medium operating voltages (~1 V) and, therefore, can be used both as cathodes [2] and anodes [3,4,5]. Titanium compounds such as TiF_3_ and TiOF_2_ have specific capacities exceeding those of carbon, possess high cycling stability and are capable to operate at high currents [3,4,5,6,7]. The latter makes them promising anode materials worthy of detailed study. The main drawbacks of these compounds are relatively low full-cell voltage and low Coulomb efficiency (CE) of the first cycle caused by a conversion reaction and formation of lithium-containing inert products unable to participate in the reversible lithiation/delithiation process. However, the mechanism of electrochemical lithiation of titanium oxyfluoride is still not fully understood, and the drawback related to the initial irreversible losses can probably be overcome by prelithiation [5].

Titanium oxyfluoride, which has semiconductor properties, finds applications in various fields including photocatalysis, UV-absorbing cosmetics, and in LIBs. It should be noted that titanium oxides and fluorides are easily converted from one to the other during the manufacturing process and, therefore, the materials under investigation are often a mixture of several compounds [8,9]. For example, TiOF_2_ heating in air leads to the formation of TiO_2_ [4] and, vice versa, fluorination of TiO_2_ (anatase) allows the formation of TiOF_2_ [5,7,10,11,12]. Dissolution of metallic titanium in HF in the presence of an oxidizer or, vice versa, in its absence, creates conditions for the formation of TiOF_2_ or, correspondingly, TiF_3_. The method of synthesis and the conditions of electrochemical tests strongly influence the value of the specific capacity, which can be reversibly stored by titanium oxyfluoride. Data available in the literature range from 220 to 526 mAh/g [3,5,7,13].

The advantage of titanium oxyfluoride in comparison with other oxides undergoing the conversion reaction during the first lithiation is that the latter proceeds with amorphization of the formed active material and is accompanied by phase separation [5]. Unlike other oxides for which, during the reverse lithium extraction process (oxidation of transition metal), the crystal structure is reduced in the reaction products, for TiOF_2_, such products remain in the amorphous phase. The latter determines a stable value of cycling capacity. The comparatively high operating potential of titanium oxyfluoride relative to lithium makes it possible to expect that lithiation at low temperatures will occur without the deposition of metallic Li, as well as in the case of lithium titanate. All this makes TiOF_2_ promising for applications as LIB anodes. Unlike other titanium compounds TiO_2_ and Li_4_Ti_5_O_12_ (LTO), the electrochemical characteristics of titanium oxyfluoride are poorly understood, and the present study aims to fill this gap.

## 2. Results and Discussion

### 2.1. Galvanostatic Tests

The electrodes were fabricated using the “slurry” technology. For this purpose, titanium oxyfluoride powders were rubbed in an agate mortar and mixed with dry components in the weight ratio T1 (or T2): carbon black: VGCF (vapor grown carbon fibers, Showa Denko) = 88%: 5%: 2%, to which a binder solution consisting of N-methylpyrrolidone and polyvinylidene fluoride (PVDF) Solef 5130 (Solvay, Shanghai, China) was then added in a 17:1 ratio. Electrodes 15 mm in diameter were mounted in CR2032 disc cells with a lithium counter electrode and Celgard 2325 as a separator. The electrolyte used was TC-E918 (Tinci), which is a 1M solution of LiPF_6_ in an EC/PC/DEC/EMC/PA mixture (ethylene carbonate, propylene carbonate, diethyl carbonate, ethyl methyl carbonate, propyl acetate).

**Figure 1:**
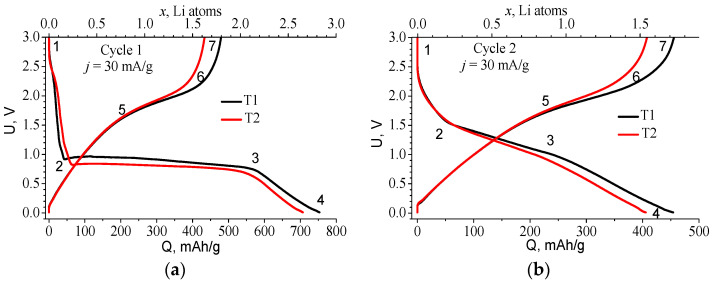
Charge–discharge curves of the T1 and T2 anodes: (**a**) first and (**b**) second cycle, recorded at *j* = 30 mA/g.

**Figure 2:**
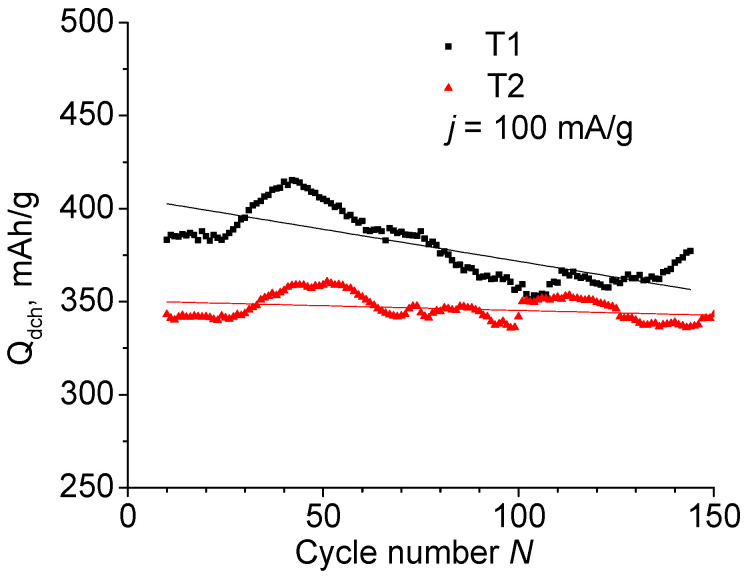
Discharge capacity of T1 and T2 anodes as a function of cycle number.

**Figure 3:**
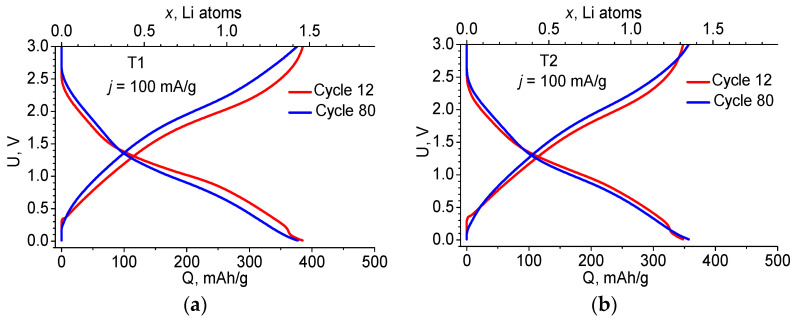
Charge–discharge curves of the 12th and 80th cycles at *j* = 100 mA/g for the T1 (**a**) and T2 (**b**) anodes.

**Figure 4:**
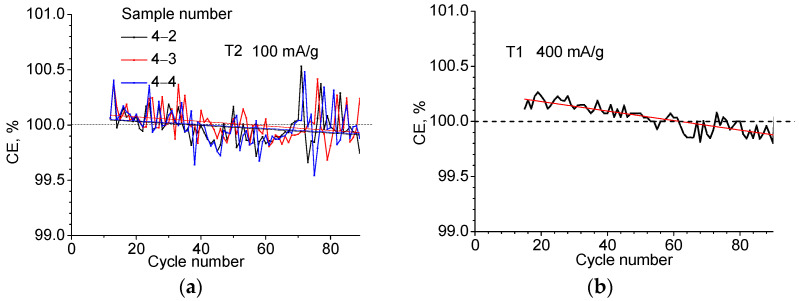
Coulombic efficiency of three T2 samples at current density *j* = 100 mA/g (**a**) and one T1 sample at *j* = 400 mA/g (**b**) as a function of cycle number.

**Figure 5:**
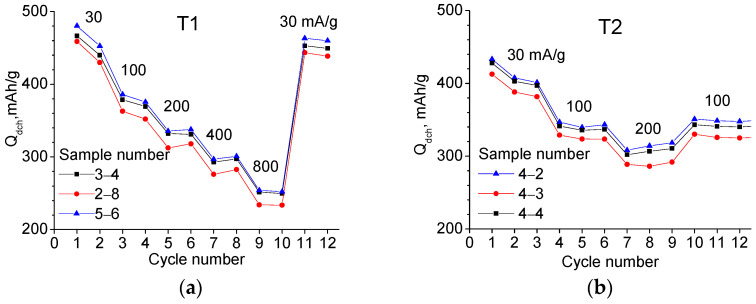
Dependencies of the discharge capacity on cycle number obtained with different charge–discharge currents shown in the figure: (**a**) for T1 and (**b**) for T2 materials (three samples of each).

Charge–discharge curves of T1-Li (or T2-Li) half-cells were recorded in galvanostatic mode using a CT3008W-5V10mA (Neware, Shenzhen, China) cycle tester. During Li embedding (charging), the voltage across the test electrodes was limited to 10 mV, and during Li extraction (discharging), it was limited to *U* = 3 V. The long-term tests, the results of which are shown in Figure 1, Figure 2, Figure 3 and Figure 4a, started with the first three cycles (*N* = 1–3) of charge–discharge being carried out at current density *j* = 30 mA/g, then three cycles (*N* = 4–6) at *j* = 100 mA/g, and another three cycles (*N* = 7–9) at *j* = 200 mA/g. Further tests were performed at *j* = 100 mA/g. Cycling at *j* = 400 mA/g, presented in Figure 4b, followed the initial 12 cycles at *j* = 30–800 mA/g (see Figure 5a). The cycling was carried out without the cell being thermostatted.

The charge curve for the first lithiation is fundamentally different from subsequent cycles (Figure 1). It has a long horizontal plateau which is characteristic of the materials forming two phases during lithiation. The position of this plateau is slightly different for the two materials: in the case of T1, it is higher with an average value of 0.82 V, and in the case of T2, with an average value of 0.74 V. Table 1 makes it possible to estimate the number of lithium g-atoms embedded in the different sections of the curve. The formula *x* = *Q*·*M*/*F* = 3.8 × 10^−3^·*Q*, where *M* is the molar mass of TiOF_2_ = 101.88 g/mol, *Q* in mAh/g, and *F* = 26,801 mAh/mol, was used for the calculation of lithium g-atoms number *x* per TiOF_2_ mole.

The structure change in TiOF_2_ as a result of electrochemical lithiation at its different stages was studied in [3,5] using X-ray diffraction (XRD), X-ray photoelectron spectroscopy (XPS), and X-ray absorption near-edge structure (XANES). According to Table 1, 0.16 and 0.24 Li atoms, respectively, are first introduced into the T1 and T2 material at voltages above the plateau. Under this stage, the structure of the material remains crystalline.

In the plateau region (range 2–3), the introduction of ~2 lithium g-atoms occurs. It is accompanied by the formation of LiF, partial reduction of Ti, and amorphization of the material [3,5]. The last lithiation range 3–4 corresponds to the introduction of Li into the amorphous phase. The maximum amount of lithium introduced during the first lithiation is 2.85 and 2.67 g-atoms for T1 and T2 materials, respectively. It can be assumed that the first lithium introduction results in a conversion reaction resulting in the formation of several compounds. It may correspond to an irreversible reaction involving 2 Li ions:

2TiOF_2_ + 2 Li^+^ + 2e^−^ → LiTiO_2_ + TiF_3_ + LiF (1)

where the oxidation state of titanium changes from 4+ to 3+. Further lithiation of titanium compounds can continue with the participation of 3 Li ions, but this time reversibly:

LiTiO_2_ + TiF_3_ + 3Li^+^ + 3e^−^ ↔ TiO + Li_2_O + Li_2_TiF_3_, (2)

which suggests two independent reactions:

LiTiO_2_ + Li^+^ + e^−^ ↔ TiO + Li_2_O (3)

TiF_3_ + 2Li^+^ + 2e^−^ ↔ Li_2_TiF_3_ (4)

The total reaction (1) and (2), corresponding to the first introduction of lithium, can be represented as:

2TiOF_2_ + 5 Li^+^ + 5e^−^ → TiO + Li_2_O + Li_2_TiF_3_ + LiF (5)

Thus, 1 mole of titanium oxyfluoride in our supposed scheme of sequential lithiation absorbs 5/2 = 2.5 lithium atom, which is in good agreement with the data in Table 1. In this case, lithium fluoride is considered to be inert, and the formation of free titanium (Ti^0^) does not occur, which corresponds to the data of [3,5]. The irreversible reaction (conversion reaction) during the first introduction of lithium into TiOF_2_ strongly reduces the Coulomb efficiency (CE) of the first cycle. For T1 and T2 materials, it is 63% and 60%, respectively. According to reactions (1)–(5), the irreversible losses of the first cycle are 2/5 = 0.4, which agrees well with the experimental values of CE. Thus, the electrochemical characteristics of both materials are extremely similar, differing only in the voltage at which the plateau appears and the value of the introduced/removed charge.

Figure 1b shows the S-curves of cycle 2, the characteristic points of these curves are given in Table 2. Starting from the second cycle a reversible redox reaction identical to the second stage of the first lithiation (2) takes place. During the lithiation, titanium is reduced to Ti^2+^ in TiO (3) and to Ti^+^ in Li_2_TiF_3_ (4), which corresponds to an average valence value of Ti^1.5+^. Hence, there are 3 Li atoms per 2 Ti atoms, i.e., Li/Ti ratio *x* = 1.5, which corresponds to a theoretical capacity of 395 mAh/g. This value is slightly lower than the total absorbed lithium *x* = 1.54 for T2 material (see Table 2) found from experiments. For T1, this value is even greater: *x* = 1.72, which can be explained by a higher content of TiF_3_. TiF_3_ can reversibly absorb 3 lithium atoms (its theoretical capacity 766.6 mAh/g) [1]. Thus, at the end of the Li embedding process, titanium is reduced to Ti^1.5+^ on average, and by the end of lithium extraction, it is oxidized to Ti^3+^. The latter is in agreement with the data of [3], which holds that the charge state of Ti decreases to values <3 during reversible lithiation, but differs from the conclusions of [5], according to which Ti^2+/4+^ reduction/oxidation occurs during lithium embedding/extraction. Since in our experiments for both materials the reversible capacity *Q*_exp_ was higher than that calculated for TiOF_2_ (within the proposed model), this discrepancy allows us to estimate the amount of TiF_3_ in each material using a simple equation:

766.6 *y* + 395(1 − *y*) = *Q*_exp_ (6)

where *y* is the mass fraction of TiF_3_. Such an estimation gives for T1 material *y* = 15.9%, and for T2, *y* = 2.7%.

### 2.2. Cycle Life

After the first nine cycles, the anodes T1 and T2 were tested at a constant current of *j* = 100 mA/g. Figure 2 shows the dependence of the discharge capacity on the number of charge–discharge cycles *N*. The observed fluctuations are due to temperature changes in the room where the tests were carried out. After ~100–140 cycles, the lithium counter-electrode degrades, which appears as a capacity drop. Replacing the lithium in the cell returns the *Q*_ch_ and *Q*_dch_ to their previous values. Fitting of the curves with linear dependence is expressed using the formulas *Q*_dch_ (T1) = 406 − 0.344*N* and *Q*_dch_ (T2) = 350 − 0.051*N*, i.e., the specific capacity of T1 material containing TiF_3_ is higher than of T2, but the degradation rate is noticeably higher, too.

Figure 3 shows how the charge–discharge curves change from cycle 12 to cycle 80 for both materials. The changes are small and are most likely due to the increase in the electrical resistance of the materials.

The Coulomb efficiency behaves somewhat unusually. From Figure 1b and Table 2, it can be seen that in the second cycle, the amount of electricity extracted exceeds the amount injected by 0.26 and 0.51% for the T1 and T2 materials, respectively, and the Coulomb efficiency appears to be >100%. Figure 4a shows the CE fluctuations occurring after the 12th cycle up to *N* = 90 when tested at constant charge/discharge current value *j* = 100 mA/g, and in Figure 4b, at *j* = 400 mA/g. Approximating the dependencies with a linear function and averaging over the samples indicates a slowly decreasing dependence with a slope of 0.002% per cycle for *j* = 100 mA/g and a slope of 0.004% for *j* = 400 mA/g. The crossing of the 100% boundary occurs between 40 and 60 cycles. It can be assumed that in this region, the degradation starts to override the effect of additional lithium release. The behavior of the Coulomb efficiency during long cycling in the galvanostatic regime seems to be a result of the fact that one of the lithium compounds formed in the conversion reaction during the first introduction of lithium (in this case LiF) does not remain completely inert and turns out to be able to contribute to the lithium yield during the reverse electrochemical reaction. The electrochemical decomposition of LiF and Li_2_O is known to be activated with the participation of transition metals or their oxides [14]. The gradual depletion of an additional source of Li occurs slowly but serves to cause the observed phenomenon where the observed CE exceeds 100% by 0.1–0.2% over several tens of cycles.

### 2.3. Power Characteristics

To investigate the charge–discharge current dependence *Q*_dch_, three samples from each material were used, which showed good reproducibility (see Figure 5). In the case of T1, the current was varied from 30 to 800 mA/g—two cycles at each current. In the case of T2, the current varied from 30 to 200 mA/g—three cycles each. In both cases, increasing the current from 30 to 200 mA/g results in a capacity reduction of 1.35 times.

### 2.4. Cyclic Voltammetry (CVA)

The measurements were carried out on a Biologic VSP modular potentiostat/galvanostat. Experiment E1 used samples that had previously undergone two cycles. In this way, the third cycle dependencies were recorded when the voltages were sweeping at different speeds (0.05–2 mV/s). In experiment E2, the sweep rate was *v* = 0.2 mV/s starting from cycle 1. In experiments E3 and E4, both materials were investigated, and the sweep speed *v* = 0.05 mV/s. These experiments make it possible to compare the electrochemical properties of T1 and T2 materials and also to determine Li diffusion coefficients in each material. Table 3 shows the details of the experiments and the parameters of the samples. The surface area *S* of the samples was calculated from the average particle size *a* in the starting material *S* = 6 *m*/(*ρ·a*); in both cases *a* = 1 μm (SEM data in figure in Section 3.2, SEM images).

**Figure 6:**
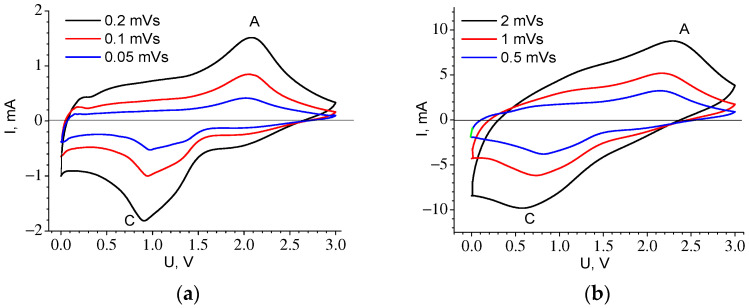
CVA recorded at different sweep speeds in T1 material (experiment E1): (**a**) *v* = 0.05–0.2 mV/s, (**b**) *v* = 0.5–2 mV/s.

The results of experiment E1 are shown in Figure 6 and Table 4.

One cathodic C and one anodic A peak are well pronounced. Their position is consistent with the flattest parts of the charge–discharge curves (see Figure 1b and Figure 3, Table 2). As the sweep speed increases, the peaks move apart in voltage from each other and become blurred.

**Figure 7:**
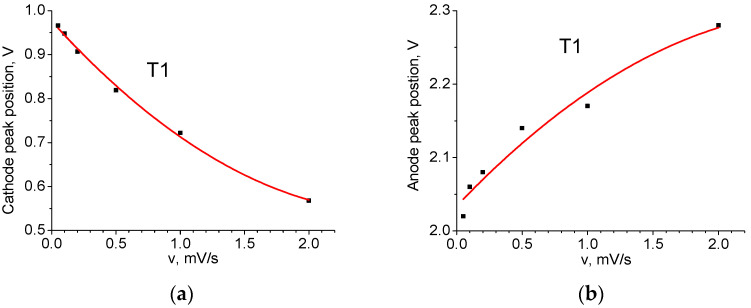
Position of voltage peaks as a function of sweep speed (T1 material): (**a**) cathode C; (**b**) anode A.

**Figure 8:**
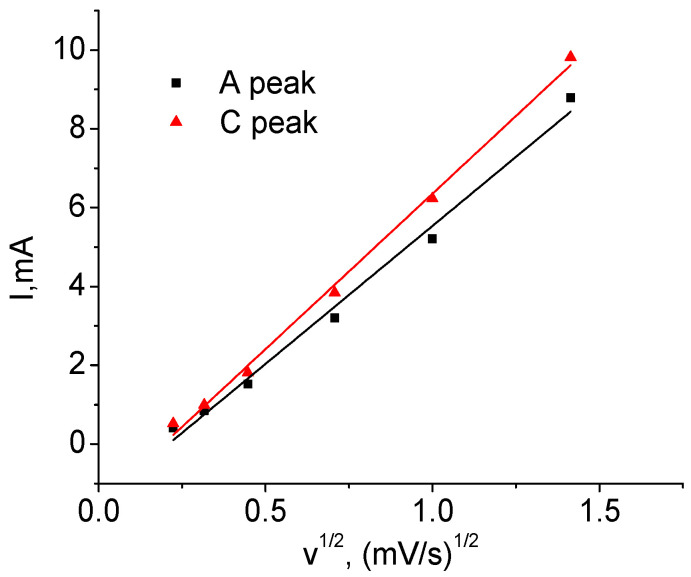
Height of the cathode and anode peaks as a function of voltage sweep speed (T1).

Figure 7 shows the dependencies of their respective voltage on *v*. The position of the peaks for *v* = 0 mV/s in Table 4 is obtained using extrapolation of the curves. It can be seen that the dependences are nonlinear, which makes it impossible to explain the shift solely by the contribution of series ohmic resistance. The cathode peak is more sensitive to the sweep speed than the anode peak: the former is shifted in voltage by 0.41 V and the latter by 0.25 V. This result indicates the asymmetry of the kinetic properties during charging and discharging. Figure 8 shows the dependence of the height of the anode (A) and cathode (C) peaks on the square root of the voltage sweep rate. Its linearity indicates that the main process limiting the charge and discharge rate is diffusion [15].

**Figure 9:**
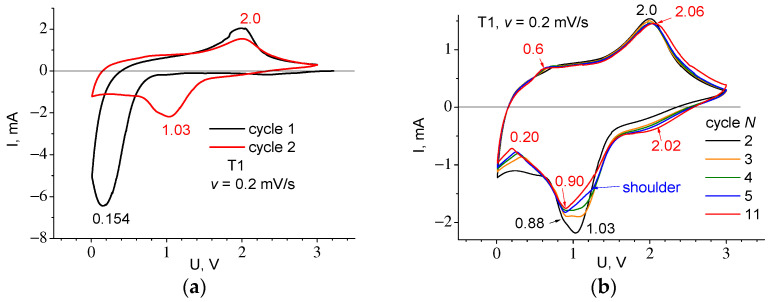
CVA curves recorded at sweep rate *v* = 0.2 mV/s for cycles: (**a**) 1 and 2 and (**b**) for 2 and 11. Cycle 2 in figures (**a**,**b**) are shown at different scales for easy comparison with other cycles.

Figure 9 shows the results of experiment two—the dependence on the number of cycles. In Figure 9b, from the intermediate cycles between the second and eleventh one, it can be seen that as the number of cycles increases, the relative height of peaks 0.88 and 1.03 is changed. This corresponds to a decrease in height of the cathode peak 1.03 with its simultaneous shift towards higher voltages so that by cycle 5, it looks like a small shoulder. At the same time, the 0.88 cathode peak and the 2 V anode peak just slightly change their voltage position. With an increasing number of cycles, an additional 0.6 V anode peak and 2.02 V cathode peak appear.

**Figure 10:**
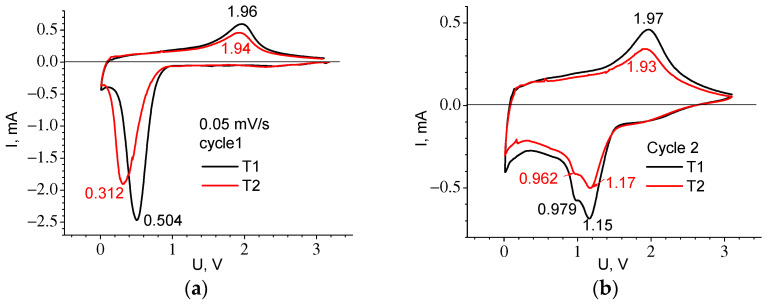
CVA curves recorded at the sweep speed *v* = 0.05 mV/s for cycle 1 (**a**) and cycle 2 (**b**).

Figure 10 shows the CVA of cycle 1 and cycle 2 recorded at low speed (experiments E3 and E4), and Table 5 shows the parameters of the observed peaks. The difference between the T1 and T2 materials is present only in the first cycle where the 1C cathode peak for T1 is located at a higher voltage than for T2, the same as the plateau in the first charging curve. The presence of two peaks on the cathodic part of the curve for cycle 2 can testify to the Li introduction mechanism corresponding to the formation of different compounds Li_2_O and Li_2_TiF_3_, (see Equations (3) and (4)). Both T1 and T2 materials exhibit significant hysteresis, which is clearly visible from the difference in voltages for the anodic and cathodic peaks (Figure 10b) and from the voltage location of the gentle sections 2–3 and 5–6 on the charge and discharge curves (Figure 1b and Table 2). In addition to the polarization component, its presence is most likely due to the so-called path hysteresis [16].

### 2.5. Determination of the Diffusion Coefficient

The mean value of diffusion coefficient *D* of lithium in the electrode material was determined using the height of CVA peaks of the dependences recorded at low speed in experiments E3 and E4 separately for the cathode and anode half-cycle. For this purpose, the Randles–Shevcik equation was applied [17,18]:

*I*_p_ = 2.69 *×* 10^5^ *S c_0_* (*z D v*)^1/2^ (7)

where *c*_0_ is the maximum concentration of lithium embedded in or extracted from the electrode in mol/cm^3^, *S* is the inner surface area [cm^2^], *v* is the voltage sweep rate [V/s], *I*_p_ is the peak current [A]. The dimension of the coefficient is 2.69 *×* 10^5^ [A·s·mol^−1^·V^−1^], and *z* is an empirical fitting parameter. Equation (7) was derived from a modified Nernst equation [15] defining the relation between the electrode potential and concentration of potential-determining particles and taking into account the difference between the concentration and activity of diffusing particles:

*E* = *E*_0_ − (*RT*/*znF*) ln *c* (8)

The necessity to introduce the fitting parameter *z* is due to the fact that the equilibrium dependence of potential *E* on *c* in the general case does not obey the Nernst equation. At room temperature, *RT*/*F* = 2.57 *×* 10^−2^ V, number of electrons *n* = 1, and therefore (8) appears as:

*E* = *E*_0_ − 2.57 *×* 10^−2^ ln *c z*^−1^ (9)

Thus, comparing the slope *s* of the empirical relationship *E* = f(ln*c*) with the slope of relationship (9) we can determine *z* = 2.57 *×* 10^−2^/|*s*|. The relation between the lithium concentration [mol/cm^3^] and the amount of electricity *Q* [C] present in the electrode is determined using the formula:

c = Q·ρ/m·F(10)

where *ρ* [g/cm^3^] and *m* [g] are the density and mass of the active component of the electrode, respectively, and the Faraday constant *F* = 9.65 *×* 10^4^ C/mol.

**Figure 11:**
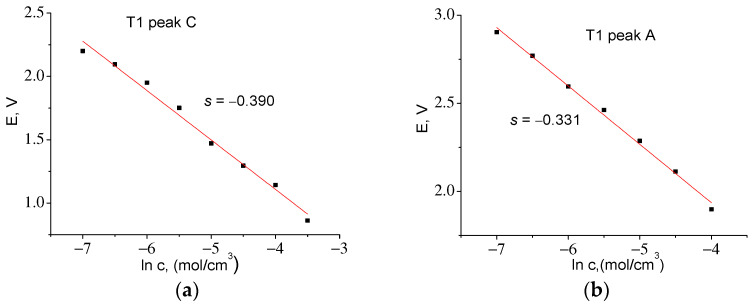
Experimental dependences of *E* = f(ln *c*) for T1 series samples for: (**a**) cathodic (*z* = 0.066) and (**b**) anodic (*z* = 0.078) parts of CVA.

Let us first determine *D* for the T1 material from CVA cycle 2 based on the height of cathode peak 3C *I*_p_ = −0.684 mA (see Table 5). To find *c*_0_, we used the maximum charge accumulated at the cathode half-cycle, *Q*_maxC_ = 12.94 C, which was obtained by integrating the dependence *I* = f(*t*). The corresponding value was *c_0_* = 0.050 mol/cm^3^. From the slope of the straight line in Figure 11a, we determined *z* = 0.066. Taking into account electrode surface area *S* = 161 cm^2^ from (7), we find *D* = 3.02 *×* 10^−14^ cm^2^/s.

**Figure 12:**
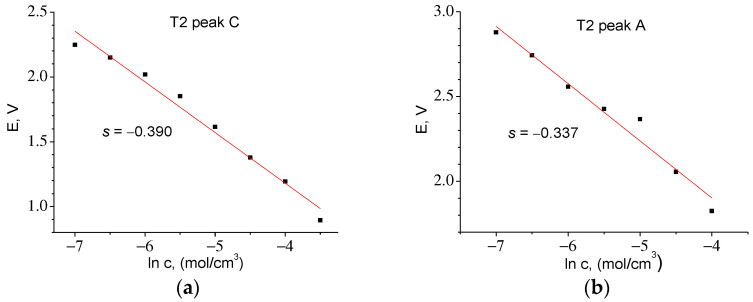
Experimental dependences of *E* = f(ln *c*) for T2 series samples: (**a**) for cathodic (*z* = 0.066) and (**b**) for anodic (*z* = 0.076) parts of CVA.

By integrating *I* = f(*t*) in the anode part of the CVA, it was found that *Q*_maxA_ = 12.43 C, *c*_0_ = 0.048 mol/cm^3^. The slope *E* = f(ln *c*) *s* = −0.331, whence *z* = 0.078 and *D* = 1.24 *×* 10^−14^ cm^2^/s. A similar procedure for the T2 material (Figure 12) made it possible to find the correction factor *z* and to calculate the diffusion coefficients for the cathode and anode peaks. Table 6 shows that the diffusion coefficients found for both materials are almost the same.

The obtained *D* values are rather low. The low Li diffusion coefficient leads to a high polarization, i.e., a large deviation in the charge and discharge dependences from the equilibrium curves. As a result, Li insertion takes place at lower potentials relative to lithium, and extraction, respectively, at higher ones. Thus, with an increase in the current, a significant reduction in capacity occurs both for Li insertion and extraction. The effect of current on the discharge capacity can be seen in Figure 5.

## 3. Materials and Methods 

### 3.1. Synthesis

The starting material was obtained using direct interaction of titanium in the air with hydrofluoric acid (48–49% HF solution in water). The condition of solid-state oxyfluoride deposition is a constant contact of titanium with the solution in which HF deficiency occurs. The reaction of titanium with hydrofluoric acid begins with the evolution of hydrogen and TiF_4_, which forms with HF in an aqueous solution a colorless fluorotitanic acid (H_2_TiF_6_). This heterogeneous reaction is exothermic and accelerates as the components heat up. At the same time, the degree of oxidation of titanium at the reaction interphase begins to decrease, apparently, due to the participation of the primary product, H_2_TiF_6_, in the further interaction. Then, along with TiF_4_, TiF_3_ is released into the HF solution giving brown-colored acid H_2_TiF_5_. As HF is exhausted, the dissociation of formed acids begins with the detachment of HF and hydrolysis of titanium tetrafluoride: TiF_4_ + H_2_O = TiOF_2_ + 2HF. TiF_3_, which is not inclined to hydrolysis, remains in the precipitate together with the released titanium oxyfluoride. When the reaction is carried out in the air, there is a possibility of gradual oxidation of finely dispersed TiF_3_ by dissolved oxygen with the formation of tetravalent titanium oxyfluoride: 4TiF_3_ + O_2_ + 2H_2_O = 4TiOF_2_ + 4HF. Therefore, without restricting air access and creating conditions for continuous close contact of titanium with HF-containing solution, it is difficult to achieve a high concentration of TiF_3_ in the resulting precipitate.

Under T1 synthesizing, when the aim was to obtain an appreciable amount of TiF_3_ in the reaction product, the process was carried out in a titanium beaker filled with small pieces of titanium and having a lid. Hydrofluoric acid was introduced in small portions with interruptions to stop active interaction with outgassing. After repeated stirring, until the visible hydrogen evolution ceased, followed by a three-hour exposure time, ethyl alcohol was poured into the beaker, and the reaction products were separated from the remaining titanium. Drying was carried out under low heat. For T2 synthesis, the reaction was carried out in an open PTFE beaker, and the products were flooded with water and separated from the excess titanium. The drying of the powder was carried out at ~70–80 °C.

### 3.2. Physicochemical Characteristics

**Figure 13:**
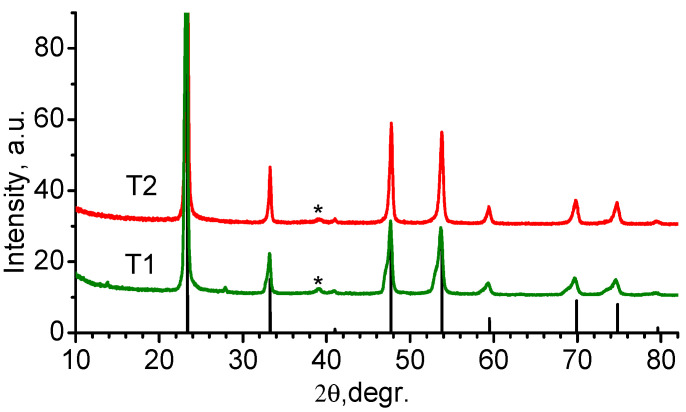
Diffraction curves from materials obtained with T1 and T2 synthesis; the line bar diagram corresponds to the positions and relative intensities of TiOF_2_ diffraction maxima PDF-01-076-7831; * peak of the most intense TiO_2_ reflex (rutile).

The obtained weakly colored powders were subjected to X-ray phase analysis (XRD). Registration of diffraction curves was carried out on X-ray diffractometer D2PHSER firm Bruker AXS (Karlsruhe, Germany) equipped with a sharp-focus X-ray tube with a copper anode (λ-k_α1_ = 1.50056 Å, k_α2_ = 1.54439 Å), PSD-detector Lynxeye. Registration of the reflected signal intensity was performed in the angle range of 10–85 degrees on the 2θ scale, the registration step was 0.02 degrees, and the signal accumulation time at the point was 1 s. Analysis of the obtained curves was carried out with the Bruker DIFFRAC.EVA V4.1.1 software package. Diffractograms were interpreted based on the ICDD database, release 2014, PDF2. The XRD results and comparison with tabulated data are shown in Figure 13.

The decoding of the diffractogram of the T2 sample showed that the main phase is the cubic modification of TiOF_2_ of space group Pm-3m. An insignificant TiO_2_ (rutile) admixture is also visible in the sample. In sample T1, the rutile admixture is also present, and the main phase is titanium oxyfluoride. However, the curve (Figure 13, T1) shows an asymmetric shape of all TiOF_2_ diffraction peaks, and the reflexes have a “shoulder” at the lower 2θ angles. This picture can be explained by the formation of an additional phase with a structure similar to titanium oxyfluoride but with a larger unit cell parameter. The lattice parameter difference between TiOF_2_ and the additional phase is 0.082 nm. This probably indicates the beginning of restructuring from TiOF_2_ to TiF_3_.

**Figure 14:**
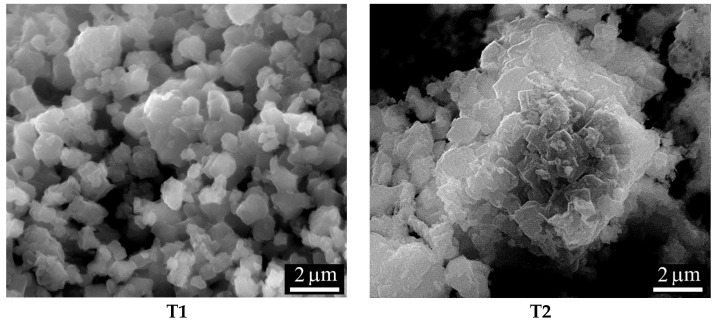
SEM images of the synthesized powders.

Electron microscopic studies and X-ray spectral microanalysis (EDX) of the obtained materials were carried out using an FEI Quanta 200 scanning electron microscope with an EDAX energy-dispersive microanalyzer. Figure 14 shows images of T1 and T2 powders, and Table 7 shows their composition. The structure of the powders can be characterized as fine-crystalline with particles~1 µm in size, on which faceting elements are visible.

The true (pycnometric) density of obtained powders was measured with gas (helium) pycnometry on an Ultrapycnometer 1000 device of Quantachrome Company (Boynton Beach, FL, USA). Table 7 shows the density of powders and their elemental composition. The latter was measured in 5 points for each powder, and the table shows average values.

It follows from the data in Table 7 that the average oxidation degree of Ti in material T1 is lower than in T2. Since the valence of Ti in the oxyfluoride must be 4, its lowering indicates a higher proportion of 3-valent titanium (TiF_3_) in the T1 material. These results are in qualitative agreement with XRD data.

## 4. Conclusions

Titanium oxyfluoride was obtained using the interaction of metallic Ti with hydrofluoric acid in a deficiency of the latter. The initial oxidation degree of titanium in the investigated products T1 and T2, formed under somewhat different conditions of reagent contact with air, was not identical. In T1, it is markedly lower than four, which is due to the presence of a larger amount of TiF_3_. The latter is in agreement with XRD data and the higher value of specific capacity of the anode.

According to the proposed model, the conversion reaction of TiOF_2_ during the first lithiation consists of irreversible and reversible parts. The former proceeds with the formation of LiTiO_2_, TiF_3_, and LiF, resulting in the reduction of titanium to Ti^3+^. The reversible redox reaction proceeds with the formation of TiO, Li_2_O, and Li_2_TiF_3_ as final reduction products and with a change in the average charge state of titanium Ti^3+/1.5+^, which corresponds to the introduction and extraction of 1.5 g-atom of lithium per 1 g-mole of TiOF_2_.

CVA studies allowed for the first time to determine the Li diffusion coefficient in titanium oxyfluoride electrodes *D* = 1.2–3.0 *×* 10^−14^ cm^2^/s.

The Coulomb efficiency of TiOF_2_ electrodes in the first cycle is low 60–63%, but in the subsequent ones, it is close to 100%. In addition, an unusual behavior of the Coulomb efficiency of TiOF_2_ exceeding 100% at cycling was revealed. The latter allows us to assume that LiF separated with the first lithiation does not remain completely inert in the subsequent delithiation processes.

Titanium oxyfluoride shows high stability in the cycling tests. During 100 cycles at a current of 100 mA/g (C/4), the T1 material retains a *Q*_dch_ higher than the theoretical value for the carbon anode. Both the T1 and T2 materials have a capacity higher than other titanium-based anodes such as TiO_2_ and LTO. T2 shows the highest degradation resistance: its discharge capacity of 345 mAh/g (*j* = 100 mA/g) practically does not change within 150 cycles.

The disadvantages of the synthesized materials are significant voltage hysteresis, which must lead to energy loss due to heat dissipation, and low Coulomb efficiency of the first cycle.

## Figures and Tables

**Table 1:** Coordinates of characteristic points on charge and discharge curves of cycle 1 (Figure 1a), their corresponding amount of embedded or extracted lithium *x*, and its increment Δ*x* at each step.

	*U*, V		*Q*, mAh/g		*x* Li		Δ*x*		
Point	T1	T2	T1	T2	T1	T2	Range	T1	T2
1	3	3	0	0	0	0			
2	0.91	0.82	42.8	62.5	0.16	0.24	1–2	0.16	0.24
3	0.73	0.66	574	557	2.17	2.11	2–3	2.01	1.87
4	0.01	0.01	753	706	2.85	2.67	3–4	0.68	0.56
5	1.69	1.62	227	199	0.86	0.76	4–5	2.23	1.91
6	2.35	2.31	442	396	1.68	1.50	5–6	0.82	0.74
7	3	3	477	432	1.81	1.64	6–7	0.13	0.14

**Table 2:** Potential and amount of lithium introduced or extracted for the characteristic points on the charge and discharge curves of cycle 2 (Figure 1b) and their corresponding *x* and increment Δ*x* at each interval between these points.

	*U*, V		*Q*, mAh/g		*x* Li		Δ*x*		
Point	T1	T2	T1	T2	T1	T2	Range	T1	T2
1	3	3	0	0	0	0			
2	1.55	1.55	52.5	56.0	0.20	0.21	1–2	0.20	0.21
3	1.01	0.96	236	220	0.89	0.83	2–3	0.69	0.62
4	0.01	0.01	454	405	1.72	1.54	3–4	0.83	0.71
5	1.79	1.69	247	221	0.94	0.84	4–5	0.78	0.70
6	2.38	2.15	414	337	1.57	1.28	5–6	0.63	0.44
7	3	3	455	407	1.73	1.55	6–7	0.16	0.27

**Table 3:** Conditions of experiments and parameters of anodes in the CVA measurement.

Experiment	Sweep Speed *v*, mV/s	Cycle Number	Material	*m*, mg	*ρ*, g/cm^3^	*S*, cm^2^
E1	0.05–2	3–9	T1	7.74	2.79	166
E2	0.2	1–11	T1	7.66	2.79	165
E3	0.05	1–2	T1	7.48	2.79	161
E4	0.05	1–2	T2	6.95	3.07	136

**Table 4:** Voltage position of C and A peaks (T1 3rd cycle).

*v*, mV/s	Peak Position Cathode, V	Peak Position Anode, V
0	0.976	2.03
0.05	0.966	2.02
0.1	0.948	2.06
0.2	0.907	2.08
0.5	0.819	2.14
1	0.722	2.17
2	0.568	2.28

**Table 5:** Parameters and designations of CVA peaks for samples T1 and T2 obtained at sweep speed *v* = 0.05 mV/s.

1 Cycle					2 Cycle				
T1		T2		Peak Designation	T1		T2		Peak Designation
*U*, V	*I*, mA	*U*, V	*I*, mA		*U*, V	*I*, mA	*U*, V	*I*, mA	
0.504	−2.46	0.312	−1.9	1C	1.15	−0.684	1.17	−0.500	3C
1.96	+0.598	1.94	+0.461	2A	0.979	−0.576	0.962	−0.414	4C
					1.97	+0.459	1.926	+0.341	5A

**Table 6:** Diffusion coefficient values for T1 and T2 electrodes (second cycle *v* = 0.05 mV/s).

	T1		T2	
	from Peak 3C	from Peak 5A	from Peak 3C	from Peak 5A
*D*, cm^2^/s	3.02 *×* 10^−14^	1.24 *×* 10^−14^	2.78 *×* 10^−14^	1.26 *×* 10^−14^

**Table 7:** Pycnometric density, the elemental composition of materials, and average oxidation degree of Ti in the studied materials T1 and T2.

	T1		T2	
Density *ρ*, g/cm^3^	2.79		3.07	
**Element**	**At%**	**Value reduced to Ti**	**At%**	**Value reduced to Ti**
Ti	25.3	1	23.9	1
O	22.6	0.89	22.6	0.94
F	52.6	2.08	53.5	2.24
Ti averaged oxidation degree	3.86		4.12	

## Data Availability

Not applicable.
